# Near-Infrared Spectroscopy for Rapid Differentiation of Fresh and Frozen–Thawed Common Carp (*Cyprinus carpio*)

**DOI:** 10.3390/s24113620

**Published:** 2024-06-04

**Authors:** Stefka Atanassova, Dimitar Yorgov, Deyan Stratev, Petya Veleva, Todor Stoyanchev

**Affiliations:** 1 Department of Agricultural Engineering, Faculty of Agriculture, Trakia University, Students Campus, 6000 Stara Zagora, Bulgaria; dimitar.yorgov@trakia-uni.bg (D.Y.); petya.veleva@trakia-uni.bg (P.V.); 2 Department of Food Quality and Safety, Faculty of Veterinary Medicine, Trakia University, Students Campus, 6000 Stara Zagora, Bulgaria; deyan.stratev@trakia-uni.bg (D.S.); todor.stoyanchev@trakia-uni.bg (T.S.)

**Keywords:** carp, fresh, frozen, near-infrared (NIR) spectroscopy, SIMCA, PLS-DA

## Abstract

This study aimed to investigate near-infrared spectroscopy (NIRS) in combination with classification methods for the discrimination of fresh and once- or twice-freeze–thawed fish. An experiment was carried out with common carp (*Cyprinus carpio*). From each fish, test pieces were cut from the dorsal and ventral regions and measured from the skin side as fresh, after single freezing at minus 18 °C for 15 ÷ 28 days and 15 ÷ 21 days for the second freezing after the freeze–thawing cycle. NIRS measurements were performed via a NIRQuest 512 spectrometer at the region of 900–1700 nm in Reflection mode. The Pirouette 4.5 software was used for data processing. SIMCA and PLS-DA models were developed for classification, and their performance was estimated using the F1 score and total accuracy. The predictive power of each model was evaluated for fish samples in the fresh, single-freezing, and second-freezing classes. Additionally, aquagrams were calculated. Differences in the spectra between fresh and frozen samples were observed. They might be assigned mainly to the O–H and N–H bands. The aquagrams confirmed changes in water organization in the fish samples due to freezing–thawing. The total accuracy of the SIMCA models for the dorsal samples was 98.23% for the calibration set and 90.55% for the validation set. For the ventral samples, respective values were 99.28 and 79.70%. Similar accuracy was found for the PLS-PA models. The NIR spectroscopy and tested classification methods have a potential for nondestructively discriminating fresh from frozen–thawed fish in as methods to protect against fish meat food fraud.

## 1. Introduction

Fish is widely consumed across the planet and is an important component of a healthy diet [1]. At the same time, fish is one of the categories of food most vulnerable to fraud. This fraud is mainly connected with species substitution, where a more expensive variety is replaced with low-value species, the mislabeling of fish regarding its geographical origin, selling thawed fish as fresh, and the undeclared use of food additives [2,3,4].

Common carp (*Cyprinus carpio*) is widely distributed and very popular in some European countries. It is one of the most frequently introduced fish species worldwide. In Europe, common carp represents more than 80% of total fish production [5]. One of the main reasons for this demand for carp is the inadequate global protein supply, and carp production could be a protein source [6]. Moreover, fresh fish is perceived as a healthier fish product than frozen ones [7].

Freezing is intensively used in preserving fish because it keeps the product safe. However, freezing changes the physical and biochemical properties of a fish’s muscle. The effects depend on the fish species, muscle type, freezing rate, storage time, storage temperature, and thawing method. Slow freezing leads to the formation of large extracellular ice crystals that damage the cell membranes and muscle proteins [8,9]. Freezing–thawing cycles are related to the degradation of textural properties due to physical damage and protein denaturation and oxidation [10].

Fresh fish is preferred by consumers who could pay more for fresh than frozen–thawed products. Fish or fish products that had been frozen before marketing must be labeled correctly. It is required according to EU regulations No. 1169/2011 and No. 1379/2013 that the preservation treatment applied must be labeled. In the case of a food product that has been frozen and then sold thawed, the name of the food must be supplemented with the term “defrosted” [11,12]. Otherwise, it is considered a fraudulent practice. Freezing after thawing or so-called second freezing is a prohibited and illegal practice that could appear at retail period. To detect and avoid such fraud, different analytical methods and approaches of analysis have been investigated and developed. These include microscopy and scanning electron microscopy histological methods [13,14], enzymatic and electrophoretic methods [15,16], and measurements of protein and lipid oxidation, volatile compounds, water-holding capacity, etc. [17]. Dielectric properties and impedance technology have been also reported for the detection of fresh and frozen–thawed seafood [18,19] and nuclear magnetic resonance spectroscopy [20].

Most of these techniques are only laboratory-applicable and not suitable for commercial applications due to the destructive nature of measurements, the time required to perform the analyses, and the cost of these analyses. There is considerable interest in the development of instrumental techniques for faster, non-destructive, and less expensive assessments of meat quality. Spectroscopic-based techniques have gained increasing attention in recent years [21]. Among them, Near-infrared (NIR) spectroscopy offers advantages over other techniques since it is non-destructive, rapid, cost-efficient, does not require chemicals to perform an analysis, and, in most cases, requires minimal sample preparation. The miniaturization of spectrometers and the development of machine learning algorithms for spectral data processing have greatly increased the further application of NIR spectroscopy.

Several authors have used NIRS and chemometrics to discriminate between unfrozen and frozen–thawed fish. Reis et al. [22] investigated possibilities for differentiation between fresh and frozen/thawed tuna fillets after a freezing period of 5, 21, and 35 days using VIS-NIR spectroscopy. Partial Least Square Discriminant Analysis (PLS-DA) was applied as a classification method. The accurate discrimination of fresh and frozen/thawed samples was reported.

The authentication of fresh West African goatfish (*Pseudupeneus prayensis*) fillets using a portable visible/near-infrared spectrometer and a compact digital camera was investigated by Ottavian et al. [23]. Excellent classification was obtained using the PLS-DA method from the VIS/NIR spectra, the color features, or a combination of spectral and color information. The most important wavelength regions for discrimination of fresh/frozen–thawed samples were found at 470, 560, 610, 930, and 970 nm. In another study, two spectral regions, NIR (1100–2500 nm) and Vis-NIR (380–1080 nm), were compared by Fasolato et al. [24] to evaluate fresh and frozen–thawed Swordfish (*Xiphias gladius* L.). The authors reported an accuracy of discrimination between fresh and frozen–thawed samples from 87.5 to 99.3% using PLSDA with cross-validation, depending on the spectral region and sample preparation—minced or whole-muscle. The Vis-NIR spectra range provided the most useful information about swordfish authentication. A handheld NIR spectrometer, working in the spectral range of 900–1700 nm for the authentication of fresh versus defrosted common white fish species using chemometric approaches, was investigated by Goncalves et al. [25]. Several machine learning algorithms were explored for classification, achieving precisions higher than 88%. Loadings from PCA revealed bands at 1150, 1200, and 1400 nm as the most discriminative spectral regions. Nieto-Ortega et al. [26] compared partial least squares discriminant models based on three non-destructive methods (bioelectrical impedance analysis, near-infrared spectroscopy, and time-domain reflectometry) to discriminate between unfrozen and frozen–thawed tuna (*Thunnus obesus*) fish. The results obtained in the evaluation of the test set were satisfactory for all the sensors, so NIR achieved the best performance (accuracy = 0.91, error rate = 0.10). Nimbkar et al. [27] reviewed novel techniques for the real-time freshness, safety, and authenticity of fish and fish products, including near-infrared spectroscopy and hyperspectral imaging in visible and near-infrared regions. Studies have shown that techniques such as machine vision and NIR spectroscopy achieve promising results in accuracy and rapidity. Nieto-Ortega et al. [28] summarized the application of near-infrared spectroscopy in the fish value chain—proximate composition, freshness, species identification, farmed vs. wild fish and types of farming, geographical origin, unfrozen vs. frozen–thawed fish, fish safety, etc. The purpose of the present study was to determine the suitability of NIR spectroscopy for the rapid, cheap, and non-destructive detection of fresh and freeze–thawed fish and propose a classification model for applications in routine fish control.

## 2. Materials and Methods

### 2.1. Samples and Experimental Design

**Figure 1:**
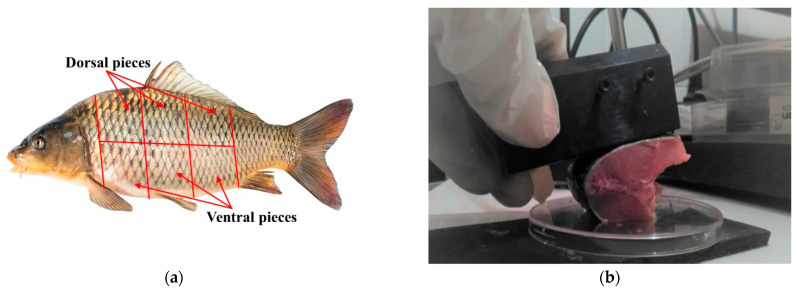
Experimental design: (**a**) scheme of cutting test pieces from dorsal and ventral regions of fish; (**b**) method of spectral measurements.

Carp (*Cyprinus carpio*) weighing 400 g to 800 g was used in the study’s experiment. The fish were delivered alive to the laboratory. Two or three test pieces (depending on the fish size) were cut from each fish’s dorsal and ventral regions in laboratory conditions (Figure 1a). A total number of 409 dorsal and 405 ventral meat pieces were prepared for NIR measurement from 140 carps. Each sample was packaged in a plastic bag before frozen storage to minimize the drying of the surface. Each fish sample was measured as fresh and after freezing in a freezer at minus 18 °C. The freezing period for the fish samples was from 15 to 20 days for the first freezing and from 15 to 21 days for the second freezing. The second freezing or refreezing of thawed fish samples was performed after the NIR measurement of the thawed first freezing samples. After the samples were removed from the freezer, they were placed in a refrigerator for 24 h at a temperature of 4 °C. After that, the samples were tempered to room temperature and measured.

### 2.2. NIR Measurements

NIRS measurements were performed using an NIR Quest 512 spectrometer (Ocean Optics, Inc., Orlando, FL, USA) in the region of 900–1700 nm with a reflection from a fiber-optics probe. The reflection fiber probe was fixed in the reflection holder to position it perpendicular to the measured surface and at a constant distance to ensure uniform measurement conditions. Each sample was measured fresh, after the first freezing and thawing, and again after the second freezing and thawing. The fish samples were measured from the skin side (Figure 1b). Several measurements at different parts of the samples were made to minimize any possible effects of structural variation in the samples.

### 2.3. Data Analysis, Classification Models, and Aquagrams

A Pirouette 4.5 (Infometrix, Inc., Bothell, WA, USA) was used to perform spectral data processing. Soft Independent Modeling of Class Analogy (SIMCA) and Partial Least Square Discriminant Analysis (PLS-DA) were used to develop the classification models. The class variable was assigned to each analyzed sample—the classes “fresh fish”, “one-time frozen/thawed”, and “two-times frozen/thawed”, respectively. The models were developed using two-thirds of the samples as a calibration data set. The rest of the samples were used as a validation data set. From the total number of 409 dorsal fish samples, 283 were used for calibration, as well as 126 for the validation data set. From the total number of 405 ventral fish samples, 280 were used for calibration, and the remaining 125 were used for the validation data set. The number of measured samples after the first and second freezing was less than that of the fresh samples because part of the samples was used for histological investigation. The same data sets were used for both methods. Separate models were developed for samples of the dorsal and ventral regions of the carp.

The SIMCA model applied was based on principal components analysis (PCA), in which the significant components of each class were evaluated using leave-one-out cross-validation. The maximum number of principal components in the SIMCA model was set to fifteen. The probability threshold was set to 0.95. The probability threshold is a value used to determine whether a sample belongs to a certain class or not.

The second classification method was PLS-DA, for which the created latent components maximized the separation between different meat samples.

The F1 score and total accuracy were used as the classification model performance indicators. The F1 score is a harmonic mean of precision and sensitivity (recall) calculated for each class. In a multi-class classification model, the F1 score for the class is a digital representation of whether the prediction on a specific class is valid. Total accuracy measures the number of correct predictions made divided by the total number of predictions made, multiplied by 100 to become a percentage.

Additionally, so-called aquagrams were calculated. An aquagram is a radar chart with coordinates related to the wavelengths of water absorption of free water and specific water configurations such as dimers, trimers, solvation shells, etc., and named water matrix coordinates [29]. The values for aquagram ${Aq}_{\lambda }$ were calculated using the following equation:

(1) $$Aq_{\lambda }=\frac{A_{\lambda }-\mu_{\lambda }}{\sigma_{\lambda }}$$

where $A_{\lambda }$ is the absorbance at wavelength λ after multiplicative scatter correction (MSC) transformation of spectral data, $\mu_{\lambda }$ is the mean value of all spectra, and $\sigma_{\lambda }$ is the standard deviation of all spectra at wavelength λ, respectively.

## 3. Results and Discussion

### 3.1. NIR Spectra of Fish Samples

**Figure 2:**
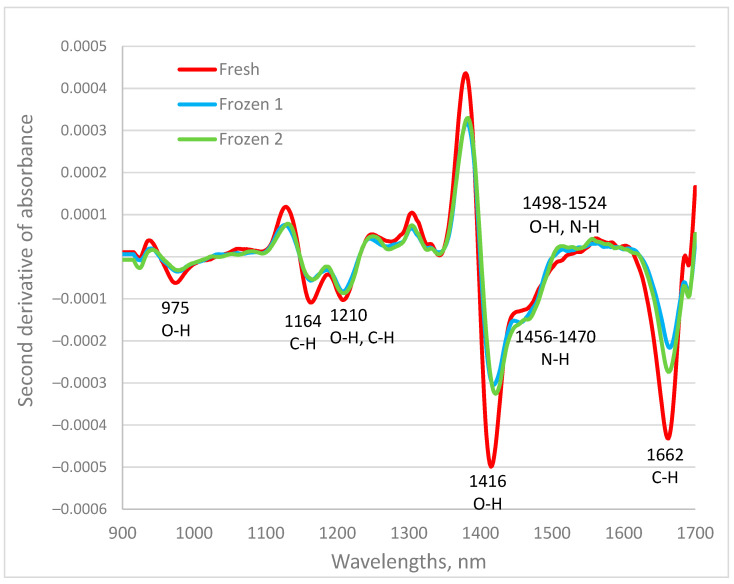
The second derivative of spectra of one fish sample from the ventral region, measured fresh, after the first freezing and thawing, and again after the second freezing and thawing.

The second derivative of fish spectra for one sample from the ventral region, measured fresh, after the first freezing and thawing, and again after the second freezing and thawing, is shown in Figure 2. The spectra were dominated by the absorption band of water in the first overtone region between 1400–1500 nm and the C-H absorption band at 1662 nm. Other weak bands were observed at 975 nm, 1164 nm, and 1210 nm. The absorption at 975 nm was related to O-H bands, at 1164 nm with C-H bands, and at 1210 nm with C-H and O-H bands, respectively. Some of these wavelengths were close to the spectral bands at 1150, 1200, and 1400 nm reported by Goncalves et al. [25] as the most discriminative spectral regions for the authentication of fresh versus defrosted common white fish species. The spectral features of carp samples reflect their chemical composition. Farmed common carp comprise approximately 70–80% water, 14–16% protein, 5–7% fat, and 1% ash, depending on age and season [30]. Water in the fish exists in the form of bound, entrapped, and free water. Most of the water in fresh fish muscle is tightly bound to the proteins and has reduced mobility. Entrapped water is another fraction of water that can be found in muscles. It can easily be converted to ice during freezing. Free water is defined as water whose flow from the tissue is unimpeded.

There were differences between the absorption patterns of fresh and frozen–thawed samples. Since the same sample was considered, the differences in the spectrum were due to the changes that occurred in it due to freezing and thawing. Freezing the fish and the thawing process changes the physical and biochemical properties of the fish’s muscle [31,32,33].

Ice crystals damage the cell membranes and distort the structure of the muscle tissue. Freezing–thawing cycles are related to conformational changes in protein molecules, denaturation and oxidation, and a decrease in the water-holding capacity of fish muscles. At the micro-level, changes lead to oxidative processes and the oxidation of lipids and proteins in destroyed cells [34,35,36]. Polyunsaturated fatty acids, which are rich in fish lipids, are prone to lipid oxidation. Moisture loss during frozen storage is another problem. Water molecules in frozen foods migrate to the surface, and the layer of ice sublimes away [37,38,39]. Myofibrillar damage depends on the size of the formed ice crystals since increasing the freeze–thaw cycles increases their size, whereas the ice structuring protein has potential as a freeze-protective component, decreasing damage and extracellular water [40]. Strateva et al. [41] reported a significant difference in the water activity of the abdominal and dorsal carp muscles after freezing. Kaltenbach et al. [20] found differences in the extracted lipid fractions and water extract of fresh and frozen–thawed mackerel, trout, and cod fish. Based on the ^1^H NMR analysis of these extracts, the authors differentiated fresh from frozen–thawed fish.

The observed spectral differences could be explained by those processes. The biggest differences in the spectra of fresh and frozen–thawed samples were observed at 1416 nm and related to the absorbance of water molecules. The intensity of absorption maxima decreased for frozen–thawed fish samples compared to the spectrum for the fresh sample. Similar observations were found for spectral maxima at 975 and 1210 nm, which were also related to water absorption. Due to the reduced water-holding capacity, after thawing, some of the water is released, and this decreases the total amount of water in the sample. Differences in spectra at 1456–1470 nm and 1498–1524 nm could be related to N-H bands of protein constituents of fish muscle. Furthermore, a small shift in the water band at 1416 nm to a higher wavelength was found for frozen samples. A similar shift in water bands in the region between 990 and 1060 nm was reported by Giró-Candanedo et al. [42] for fresh and frozen–thawed mackerel fish. There were small differences between spectra of the same sample measured after the first and second freezing–thawing cycles.

**Figure 3:**
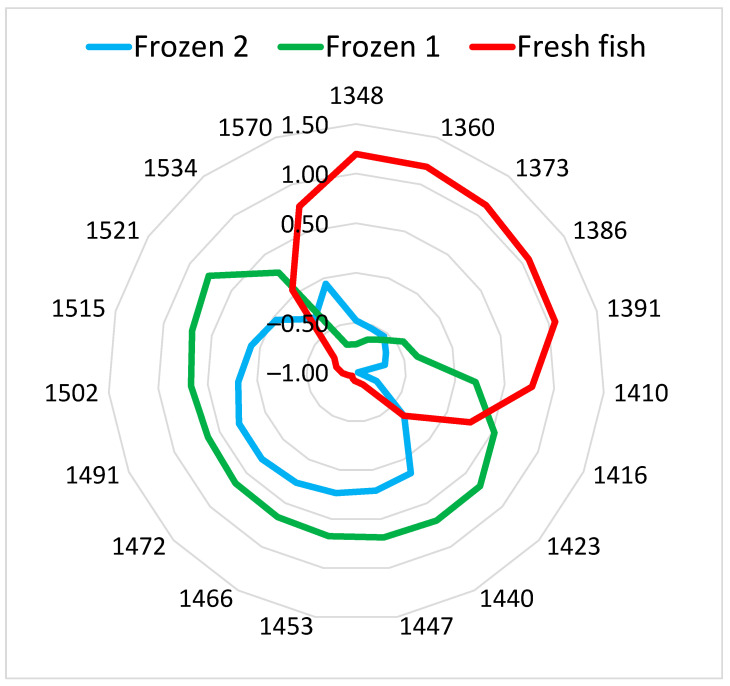
Aquagram of fish samples, measured initially fresh and then after the first and second freezing–thawing cycles.

To visualize in detail the changes in the water absorbance pattern, a chart termed an “Aquagram” was used. The aquagram, calculated using spectral data from the same fish sample, initially measured fresh and then after the first and second freezing–thawing cycles, is presented in Figure 3.

The aquagram pattern of the fish sample after freezing changed significantly. The aquagram values of the frozen samples decreased from 1347 to 1409 nm and increased in the range of 1422 to 1521 nm. Similar aquagram patterns were found for the rest of the samples. The region between 1350 and 1400 nm included the absorbance of weakly hydrogen-bonded water and trapped water. The region of 1450–1550 nm was related to strongly bound water molecules and water with two, three, and four hydrogen bonds [29,43]. Freezing increased the concentration of dissolved materials. The changes in ionic concentration affected the hydrogen bonding of the water and the formation of hydrogen bonds between oxygen or nitrogen in the ions and water. The aquagrams clearly showed changes in the ratio of free water to bound water and the number of hydrogen bonds between water molecules in frozen–thawed fish compared to fresh fish.

### 3.2. SIMCA Classification of Fresh and Frozen Fish Samples

It was not possible to distinguish the fish samples as fresh or frozen–thawed using raw spectral data. SIMCA models for the discrimination of fresh, once-frozen–thawed, and twice-frozen–thawed fish were developed separately for dorsal and ventral fish pieces. The performance of the SIMCA models was compared in terms of the F1 score and total accuracy. The best models were obtained using the second derivative transformation of spectral data.

**Figure 4:**
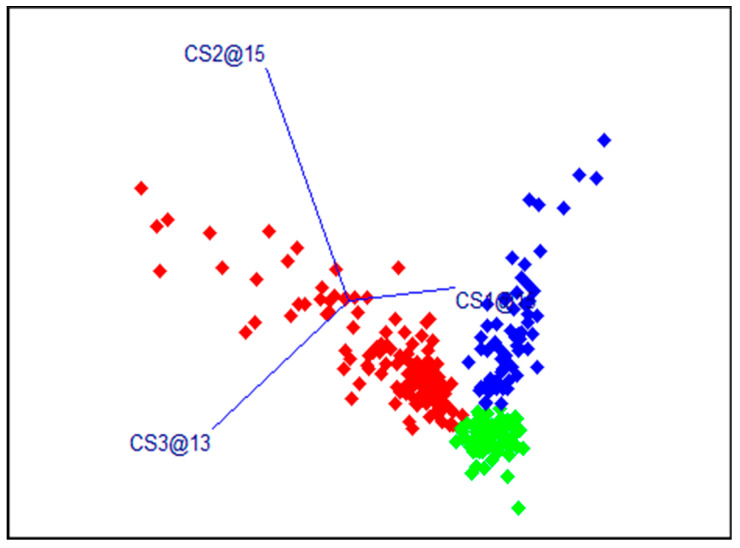
Results of the SIMCA models, dorsal region, and calibration data set. In red—fresh samples, green—samples after a single freezing, and blue—samples after a double freezing. The designations of the coordinate axes CSx@xx show how many PC factors were included in the SIMCA model for the corresponding class.

The results of the SIMCA models for dorsal samples with the calibration data set are presented in Table 1 and Figure 4. The total accuracy of the calibration models was 98.2%. Table 2 presents the results of the SIMCA models in predicting the class values of samples from the independent validation set. Most of the samples were correctly classified. The total accuracy was 90.7%, and F1 scores varied from 84.4 to 95.3%.

**Figure 5:**
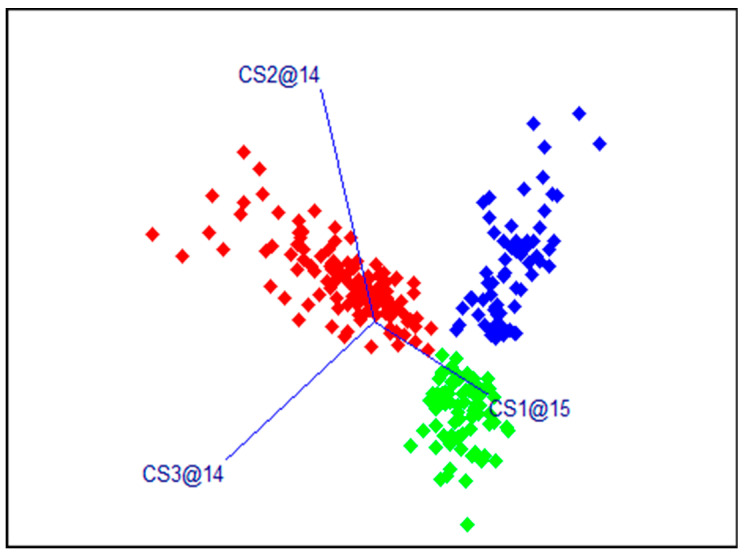
Results of the SIMCA models, ventral region, and calibration data set. In red—fresh samples, green—samples after a single freezing, and blue—samples after a double freezing. The designations of the coordinate axes CSx@xx show how many PC factors were included in the SIMCA model for the corresponding class.

The SIMCA models, developed using spectra of samples from the ventral fish region, exhibited similar performance. The total accuracy of the calibration models was 99.2%, and the F1 score ranged from 98.8 to 99.6% (Table 3 and Figure 5). When the SIMCA model was validated with the independent sample set, the total accuracy was 79.7%, and the F1 score was 86.5% for the recognition of fresh samples, 76.0% for single-frozen, and 82.4% for double-frozen (Table 4). One sample from the single-frozen group was predicted as “No match”, which means the sample was not assigned to any of the predefined classes.

When comparing the accuracy of the obtained models for the two investigated parts of the fish—dorsal and ventral—slightly better accuracy in classification was obtained for the dorsal part. In all cases, for the validation data set, the F1 score was high for the group of fresh fish samples—95.3% for the dorsal region and 86.5% for the ventral region. The accuracy of the models in determining samples after a second freeze–thaw cycle was better than that for samples frozen only once. This indirectly indicates that the changes in the samples and the obtained spectra after the second freezing were larger than after the first freezing. This confirms the results of Popelka et al. [44] in the histological investigation of fresh, frozen, and double-frozen rainbow trout. The authors reported that, in once-frozen fish, mild damage to the muscle fibers was demonstrated, but in double-frozen fish, intense damage to the muscle fiber structure and the total disruption of muscle fibers were observed. Strateva et al. [41], in a histological study of fresh and frozen carp (*Cyprinus carpio*), reported clear morphological differences between the muscle fibers of fresh carp and carp frozen once or twice. Muscles were damaged to a greater extent after double freezing.

### 3.3. PLS-DA Classification of Fresh and Frozen Fish Samples

The results of the PLS-DA models for dorsal samples were presented in Table 5 for the calibration data set and Table 6 validation data set. The total accuracy of the calibration model was 97.5%. The performance accuracy of the model using an independent data set was 81.0%, and F1 scores varied from 78.6 to 87.5%.

The PLS-DA models for the samples from the ventral region had slightly higher accuracy than those for the dorsal samples (Table 7 and Table 8). The total accuracy of the calibration model was 98.2%, and for the validation data set, the corresponding value was 83.2%. The F1 scores for the validation data set were 89.6% for the fresh carp samples, 82.7 for the single-frozen–thawed samples, and 88.2% for the double-frozen–thawed samples.

The results obtained from the investigation were similar to those reported by other authors who used PLS-DA as a classification method. The accuracy of discrimination between fresh and frozen–thawed samples from 87.5 to 99.3% using PLS-DA with cross-validation was reported by Fassolato et al. [24]. Using VIS-NIR spectra for the differentiation between fresh and frozen/thawed tuna fillets, Reis et al. [22] reported 88% total accuracy, 82% sensitivity, and 93% specificity. Precision higher than 88% for the differentiation of fresh and defrosted common white fish species was reported by Goncalves et al. [25] using a handheld NIR spectrometer in the same spectral range of 900–1700 nm. Giró-Candanedo et al. [42] investigated the discriminative power of low-cost NIR devices and PLS-DA in classification models for fresh and thawed fish samples with a rate for fresh fillets of 90.3% and 94.1% and for frozen samples of 91.2% and 89.7%, respectively. Kaltenbach et al. [20], on the base of the ^1^H NMR analysis of the lipid fraction of fish and PLS-DA, differentiated fresh from frozen–thawed fish and reported a correct prediction of 90%.

If we compare the models obtained via the two methods used—SIMCA and PLS-DA—the biggest difference is in the number of samples that are not explained via the models. Fish samples that were not classified via the models as “fresh”, a “single freezing” or a “second freezing” were placed in the unknown “No match” class category. In the case of the SIMCA models, there was the absence of “no match” samples during the calibration data set. In the validation data set, “no match” samples represented only one of the samples from the dorsal region and eight samples from the ventral region. When PLS-DA was used as a classification method, more samples were unmodeled. Six samples from the dorsal region and five samples from the ventral region were unmodeled for the calibration data set, as well as eight and eleven samples in the validation data set, respectively.

## 4. Conclusions

There are clear differences between the absorption spectra in the near-infrared region from 900 to 1700 nm in fresh and frozen–thawed carp. The spectral differences could be explained by changes in chemical composition due to the damaging of cell membranes via ice crystals during freezing and subsequent thawing, proteolysis and the modification of muscle lipids, and changes in free and bound water.

Both of the classification methods used—SIMCA and PLS-DA—allowed for the discrimination of fresh from frozen–thawed fish with an accuracy higher than 78.5% using measurement samples directly from the fish surface.

NIR spectroscopy has potential as a fast, non-destructive, valuable method for detecting fraud or the mislabeling of frozen–thawed fish or meat sold as fresh.

## Figures and Tables

**Table 1:** Performance of SIMCA model for dorsal region—calibration data set.

	Determined as Fresh	Determined as Single-Frozen	Determined as Double-Frozen	F1 Score, %
Fresh fish	130	4	0	98.5
After a single freezing	0	84	0	97.1
After a second freezing	0	1	64	99.2

**Table 2:** Performance of SIMCA models for dorsal region—validation data set.

	Determined as Fresh	Determined as Single-Frozen	Determined as Double-Frozen	No Match	F1 Score, %
Fresh fish	51	2	0	0	95.3
After a single freezing	3	35	1	1	84.4
After a second freezing	0	4	29	0	90.6

**Table 3:** Performance of SIMCA model for ventral region—calibration data set.

	Determined as Fresh	Determined as Single-Frozen	Determined as Double-Frozen	F1 Score, %
Fresh fish	133	1	0	99.6
After a single freezing	0	83	0	98.8
After a second freezing	0	1	62	99.2

**Table 4:** Performance of SIMCA models for ventral region—validation data set.

	Determined as Fresh	Determined as Single-Frozen	Determined as Double-Frozen	No Match	F1 Score, %
Fresh fish	48	0	0	5	86.5
After a single freezing	4	30	4	1	76.0
After a second freezing	1	2	28	2	82.4

**Table 5:** Performance of PLS-DA models for the dorsal region—calibration data set.

	Determined as Fresh	Determined as Single-Frozen	Determined as Double-Frozen	No Match	F1 Score, %
Fresh fish	128	0	0	6	97.7
After a single freezing	0	84	0	0	99.7
After a second freezing	0	1	64	0	99.2

**Table 6:** Performance of PLS-DA models for the dorsal region—validation data set.

	Determined as Fresh	Determined as Single-Frozen	Determined as Double-Frozen	No Match	F1 Score, %
Fresh fish	41	9	2	1	85.4
After a single freezing	2	33	1	4	78.6
After a second freezing	0	2	28	3	87.5

**Table 7:** Performance of PLS-DA model for the ventral region—calibration data set.

	Determined as Fresh	Determined as Single-Frozen	Determined as Double-Frozen	No Match	F1 Score, %
Fresh fish	129	0	0	4	98.5
After a single freezing	0	80	0	1	99.4
After a second freezing	0	0	66	0	100.0

**Table 8:** Performance of PLS-DA model for the ventral region—validation data set.

	Determined as Fresh	Determined as Single-Frozen	Determined as Double-Frozen	No Match	F1 Score, %
Fresh fish	43	3	1	6	89.6
After a single freezing	0	31	4	4	82.7
After a second freezing	0	2	30	1	88.2

## Data Availability

All data are available from the corresponding author upon request.
